# A bibliometric analysis of the role of microbiota in trauma

**DOI:** 10.3389/fmicb.2023.1091060

**Published:** 2023-02-02

**Authors:** Runzhi Huang, Yuwei Lu, Minghao Jin, Yifan Liu, Mengyi Zhang, Shuyuan Xian, Zhengyan Chang, Lei Wang, Wei Zhang, Jianyu Lu, Xirui Tong, Siqiao Wang, Yushu Zhu, Jie Huang, Luofeng Jiang, Minyi Gu, Zongqiang Huang, Minjuan Wu, Shizhao Ji

**Affiliations:** ^1^Department of Burn Surgery, The First Affiliated Hospital of Naval Medical University, Shanghai, China; ^2^Research Unit of Key Techniques for Treatment of Burns and Combined Burns and Trauma Injury, Chinese Academy of Medical Sciences, Beijing, China; ^3^Shanghai Jiao Tong University School of Medicine, Shanghai, China; ^4^Department of Orthopedics, The First Affiliated Hospital of Zhengzhou University, Zhengzhou, China; ^5^Department of Pathology, Shanghai Tenth People's Hospital, Tongji University of Medicine, Shanghai, China; ^6^Beijing Genomics Institute (BGI), Shenzhen, China; ^7^Tongji University School of Medicine, Shanghai, China

**Keywords:** gut microbiota, trauma, bibliometrics, bibliometrix, inflammation

## Abstract

**Introduction:**

Over the last several decades, the gut microbiota has been implicated in the formation and stabilization of health, as well as the development of disease. With basic and clinical experiments, scholars are gradually understanding the important role of gut microbiota in trauma, which may offer novel ideas of treatment for trauma patients. In this study, we purposed to summarize the current state and access future trends in gut microbiota and trauma research.

**Methods:**

We retrieved relevant documents and their published information from the Web of Science Core Collection (WoSCC). Bibliometrix package was responsible for the visualized analysis.

**Results:**

Totally, 625 documents were collected and the number of annual publications kept increasing, especially from 2016. China published the most documents while the USA had the highest local citations. The University of Colorado and Food & Function are respectively the top productive institution and journal, as PLOS One is the most local cited journal. With the maximum number of articles and local citations, Deitch EA is supported to be the most contributive author. Combining visualized analysis of keywords and documents and literature reading, we recognized two key topics: bacteria translocation in trauma and gut microbiota's effect on inflammation in injury, especially in nervous system injury.

**Discussion:**

The impact of gut microbiota on molecular and pathological mechanism of inflammation is the focus now. In addition, the experiments of novel therapies based on gut microbiota's impact on trauma are being carried out. We hope that this study can offer a birds-eye view of this field and promote the gradual improvement of it.

## 1. Introduction

Trauma, a kind of injury to living tissues caused by extrinsic agents with high morbidity involving fracture, various visceral injuries, and so on, is one of the major contributors to mortality. According to the National Center for Trauma Medicine in China, over 62 million people seek treatment for trauma annually in China. According to WHO (/www.who.int/data/gho/data/themes/mortality-and-global-health-estimates), injuries killed 4.4 million people worldwide in 2019 due to both incidental and deliberate causes, accounting for 8% of all deaths. Although, on account of the COVID-19 pandemic, unintentional injuries ranked fourth in 2020, down one place from the past 5 years in the USA, the mortality statistics were still horrendous (Ahmad and Anderson, 2021). The common causes of death in trauma patients include hemorrhage, sepsis, multiple organ failure (MOF) and central nervous system injury. Aside from mortality, incapacity and organic damage induced by trauma, which significantly reduce the quality of patients' lives, are also issues that increase family and social expenses. It has been assessed that the global economic loss caused by traumatic brain injury alone is nearly 400 billion dollars per year (Maas et al., 2017). Consequently, improving the management and treatment of patients in trauma to boost survival and prognosis is crucial. What's more, the pathophysiological mechanism of trauma which is vital for therapy studies, is not completely clear and requires more researches.

Gut microbiota refers to the microorganisms which inhabit digestive tracts, covering bacteria, fungi, viruses and archaea, etc. It is the largest community of commensal microbes in the human body, predominantly composed of bacteria, as the bacteria-to-host-cell ratio is significantly closer to 1:1 (Sender et al., 2016). With the dominant contribution of bacteria, the quantity of gut microbial genes in a single person reaches 3.3 million, nearly 150 times the size of human genomes, showing the genetic potential of gut microbiota to impact human health (Qin et al., 2010). Actually, in recent years, numerous studies and evidences in various levels have demonstrated that these gut microorganisms play a number of important roles in human body, including modulating the functions of endocrine system (Qi et al., 2021), immune system (Round and Mazmanian, 2009; Maranduba et al., 2015; Bailey and Cryan, 2017), and nervous system (Dalile et al., 2019), and inflammatory response (Al Bander et al., 2020). Thus, imbalance of gut microbiota is related to various enteral and parenteral diseases, such as inflammatory bowel disease (IBD) (Lavelle and Sokol, 2020), type 2 diabetes (Gurung et al., 2020), Alzheimer's disease (Kesika et al., 2021), and depression (Simpson et al., 2021). The effect of intestinal microbiota in the treatment of diseases is attracting increasing attention as well.

As gut-origin infection is not uncommon in patients with injury and gut bacterial translocation has been found in trauma (Deitch et al., 1987), scholars have hypothesized that gut microbiota participates in the pathophysiologic mechanism of trauma and is related to patients' prognosis. For instance, many scholars demonstrated that the disorder of brain-gut axis plays a key role in neuroinflammation of nervous system injuries like stroke (Singh et al., 2016), traumatic brain injury (Sundman et al., 2017; Hanscom et al., 2021) and spinal cord injury (Wallace et al., 2019; Jing et al., 2021). There's also evidence that gut microbiota can influence outcomes of ischemic brain injury by regulating intestinal regulatory T (Treg) cells and IL-17+ γδ T cells, altering immune homeostasis (Benakis et al., 2016). Scholars have been trying to find the specific mechanism through which patients' own gut microbiota impacts the development of trauma, which may ultimately provide a new therapeutic direction to improve patients' outcomes.

Bibliometrics is a set of mathematical and statistical methods to evaluate literature, journals, authors of these articles and other published works quantitatively in different fields (Jones, 2016). With bibliometric analysis, scholars can overview the hotspots and estimate the development tendency in a scientific field. So far, this convenient analytical approach has been applied in numerous medical fields, comprising obstetrics and gynecology (Brandt et al., 2019), radiology (Bluemke, 2020) and periodontology (Ahmad and Slots, 2000). Nevertheless, bibliometric analysis in the field of gut microbiota and trauma remains a void. Therefore, it's meaningful to conduct the bibliometric study to comprehensively analyze the literature on the relationship between gut microbiota and trauma, exploring the field structure, current state and trend topics. We hope that our results will serve as a reference for future studies in the field.

## 2. Materials and methods

### 2.1. Data sources and research strategies

By using Web of Science Core Collection (WoSCC), we performed a data search related to gut microbiota and trauma, retrieving SCI documents with no language limitation. The research strategy we designed was [(TS = trauma) OR (TS = traumatic)] AND [(TS = gut) OR (TS = intestin*) OR (TS= gastrointestin*) OR (TS = gastro-intestin*)] AND [(TS = microbiot*) OR (TS = microbiome*) OR (TS = flora) OR (TS = microflora) OR (TS = bacteria) OR (TS = prebiotic) OR (TS = probiotic)]. We conducted the retrieval on May 15, 2022. After excluding reviews and books and restricting the scope to “article”, the information of all the remaining 625 relevant articles, which we analyzed subsequently, was downloaded as TXT files (Peng et al., 2022). All the retrieval works and data collection were accomplished on May 15, 2022.

### 2.2. Data analysis

The TXT files were imported into Biblioshiny, a web application for a bibliometric tool developed in R version 4.2.0 (Institute for Statistics and Mathematics, Vienna, Austria; www.r-project.org) (Aria and Cuccurullo, 2017). The application was mainly employed to visualize sources, authors, and documents' analysis and present the conceptual, intellectual, and social structure, with the bibliometrix package. Besides, we utilized CiteSpace software version 6.1.2R, a computational tool based on JAVA for data visualization (Chen, 2004), and VOSviewer version 1.6.18, a bibliometric program for constructing visualized networks (van Eck and Waltman, 2010), to repeat the process and validate the results.

Annual scientific production and average citations were acquired to analyze the overall trend in this field. The impactive countries, institutions, journals and authors were mostly estimated by their production and citations. The algorithms like Bradford's law (Brookes, 1969), Lotka's law (Pao, 1985) and h-index (Hirsch, 2005) were applied to find the core journals and authors. We also visualized the collaboration of countries to assess the global relationship between countries. In order to detect the hotspots, after capturing high-frequency keywords and important documents and building networks of co-word and direct citations, we accessed the concrete content of some relevant articles in this field. The trend topic in this field was obtained from the trend topics map and direct citation network.

## 3. Results

### 3.1. Annual publication and citation

**Figure 1:**
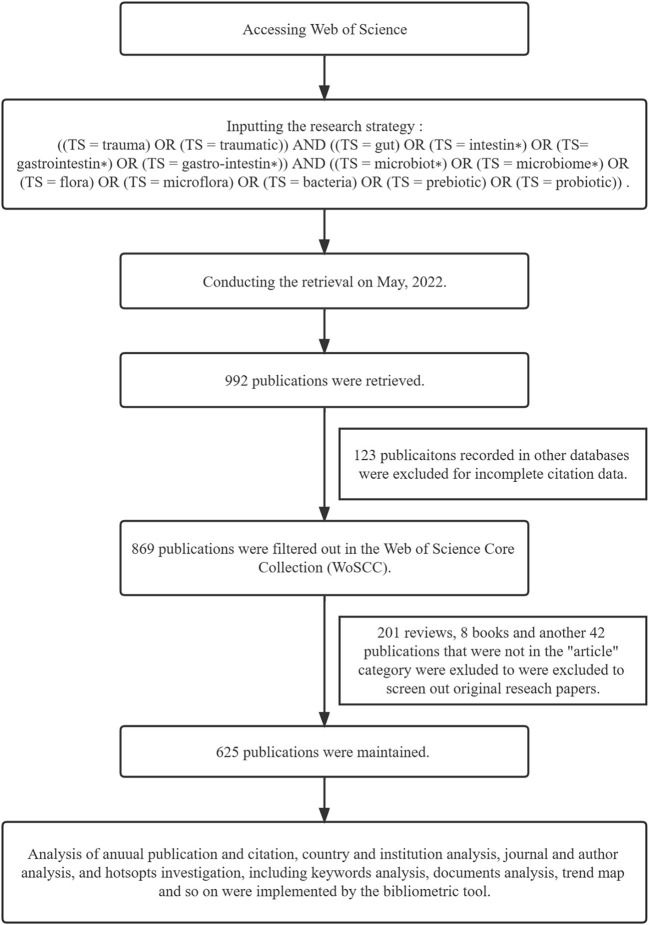
The retrieval flow chart.

**Figure 2:**
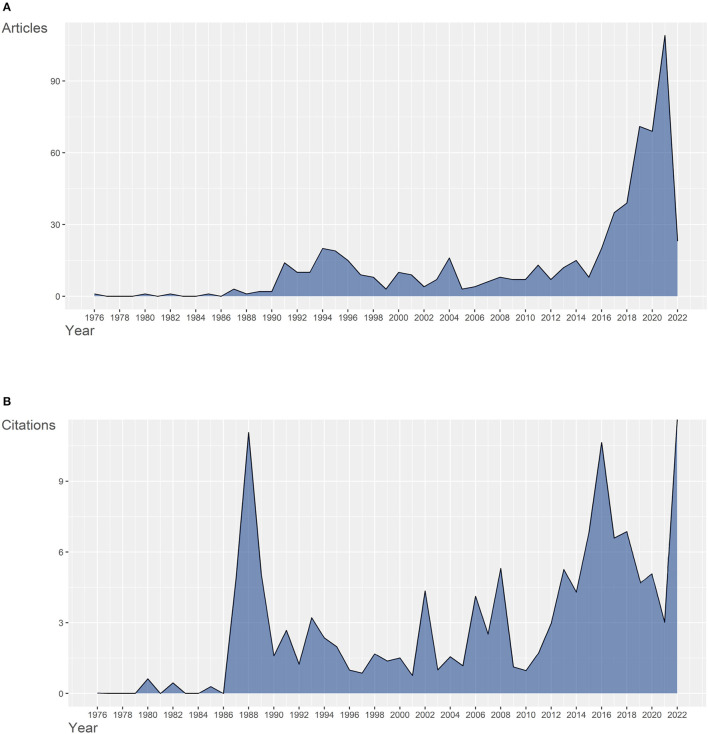
**(A)** Quantity of annual scientific production in gut microbiota and trauma research from 1976 to 2022. **(B)** Citations of average article per year in gut microbiota and trauma research from 1976 to 2022.

Based on the retrieval process shown in Figure 1, 625 relevant articles were collected from WoSCC. The first SCI publication with complete citation information in gut microbiota and trauma research, was found to published in 1976. Figure 2A presents the number of published documents per year. The quantity of production showed no obvious change until the first small peak in 1987. In 1991, the number of documents published per year increased to 14. It's noteworthy that the manifestly explosive growth started from 2016 and didn't reach the peak, pointing out that scholars made scientific breakthroughs or more research directions were detected. The growing trend indicated the role of gut microbiota in trauma were gaining increasing traction and had great development potential in both clinical and basic experiments.

The statistics of annual article citations per year were revealed in Figure 2B. The two peaks, respectively, occurred in 1988 and 2016, implicating that important research outcomes were gained. More information of documents analysis is included in point 3.4.3.

### 3.2. Most influential country and institution

**Figure 3:**
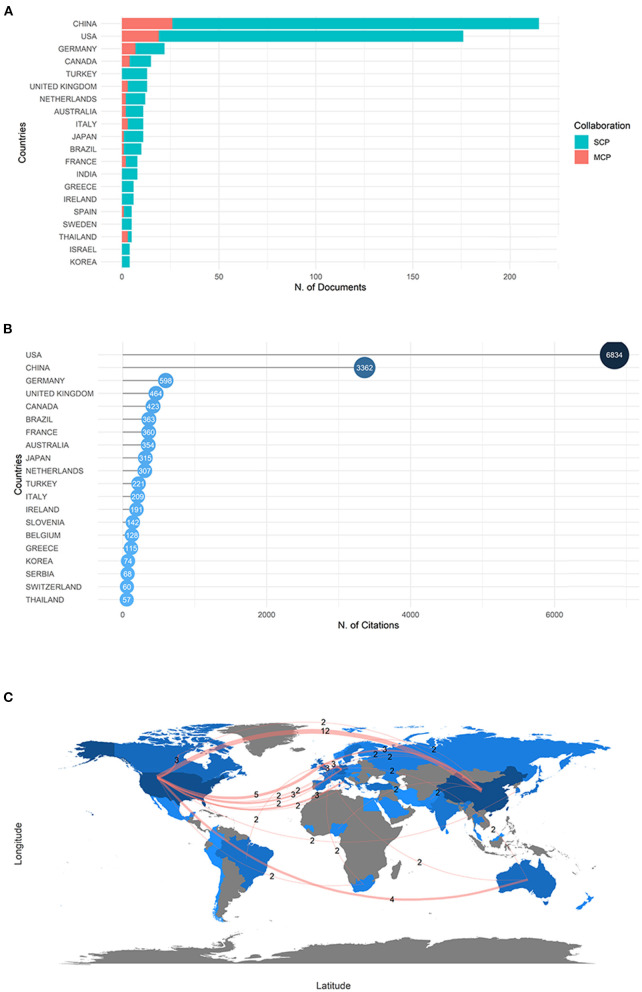
**(A)** Top 20 countries production and collaboration histogram based on the nationality statistics of the corresponding authors in gut microbiota and trauma studies. SCP, single country publications. MCP, multiple country publications. **(B)** Top 20 most cited countries in gut microbiota and trauma research. **(C)** Country collaboration map in gut microbiota and trauma research.

Forty-nine countries took part in the field of gut microbiota and trauma, spanning from 1976 to 2022. The map developed by Biblioshiny depicted the distribution and quantity of countries of publication (Figure 3A). The frequency of publication in these countries was based on the nationality statistics of the corresponding authors. China was the most prolific country (*n* = 215), followed by the USA (*n* = 176) and German (*n* = 22) (Table 1), illustrating the absolutely leading position of China and the USA in terms of publication volume. However, with high quantity of production, the number of the citation of China was nearly half of the USA (Figure 3B), emphasizing the powerful influence of the USA in this field. In the collaboration analysis, we set the minimum edge to two and labeled the numbers of collaborations on the map (Figure 3C). According to the country collaboration map, the USA had the most ties with other major publishing countries. There were 130 collaborations between countries worldwide, 51 of which were between the USA and other countries. The line between the USA and China was widest, highlighting the association between the two countries for gut microbiota and trauma research. As a whole, the link lines between countries were sparse and scattered, which corresponds with the information that the single country publications were much more than the multiple country publications (Table 1). These results suggested that cooperation among countries should be promoted.

**Table 1:** Top 20 countries production and collaboration based on the nationality statistics of the corresponding authors in gut microbiota and trauma research.

**Rank**	**Country**	**Articles**	**Proportion of articles (%)**	**SCP**	**MCP**	**Proportion of MCP (%)**
1	China	215	36.50	189	26	12.09
2	USA	176	29.88	157	19	10.80
3	Germany	22	3.74	15	7	31.82
4	Canada	15	2.55	11	4	26.67
5	Turkey	13	2.21	13	0	0.00
6	United Kingdom	13	2.21	10	3	23.08
7	Netherlands	12	2.04	10	2	16.67
8	Australia	11	1.87	9	2	18.18
9	Italy	11	1.87	8	3	27.27
10	Japan	11	1.87	10	1	9.09
11	Brazil	10	1.70	9	1	10.00
12	France	8	1.36	6	2	25.00
13	India	8	1.36	8	0	0.00
14	Greece	6	1.02	6	0	0.00
15	Ireland	6	1.02	6	0	0.00
16	Spain	5	0.85	4	1	20.00
17	Sweden	5	0.85	5	0	0.00
18	Thailand	5	0.85	2	3	60.00
19	Israel	4	0.68	4	0	0.00
20	Korea	4	0.68	4	0	0.00

SCP, single country publications; MCP, multiple country publications.

**Figure 4:**
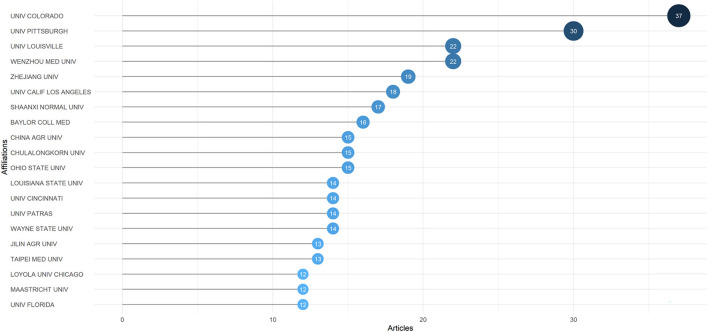
Top 20 most productive institutions in gut microbiota and trauma research.

According to our retrieved data, 878 institutions participated in the research of gut microbiota and trauma. We listed the top 20 prolific institutions (Figure 4). It's not surprising that three of the top three institutions, including the University of Colorado (*n* = 37), the University of Pittsburgh (*n* = 30), and the University of Louisville (*n* = 22), were located in the USA. Another institution tied for third was Wenzhou Medical University, which was from China. Except for Chulalongkorn University (Thailand, *n* = 15), University of Patras (Greece, *n* = 14) and Maastricht University (Holland, *n* = 12), other institutions were from either the USA or China, proving remarkable achievement of the two countries in this field again.

### 3.3. Most impactive journals and authors

#### 3.3.1. Most impactive journals

**Figure 5:**
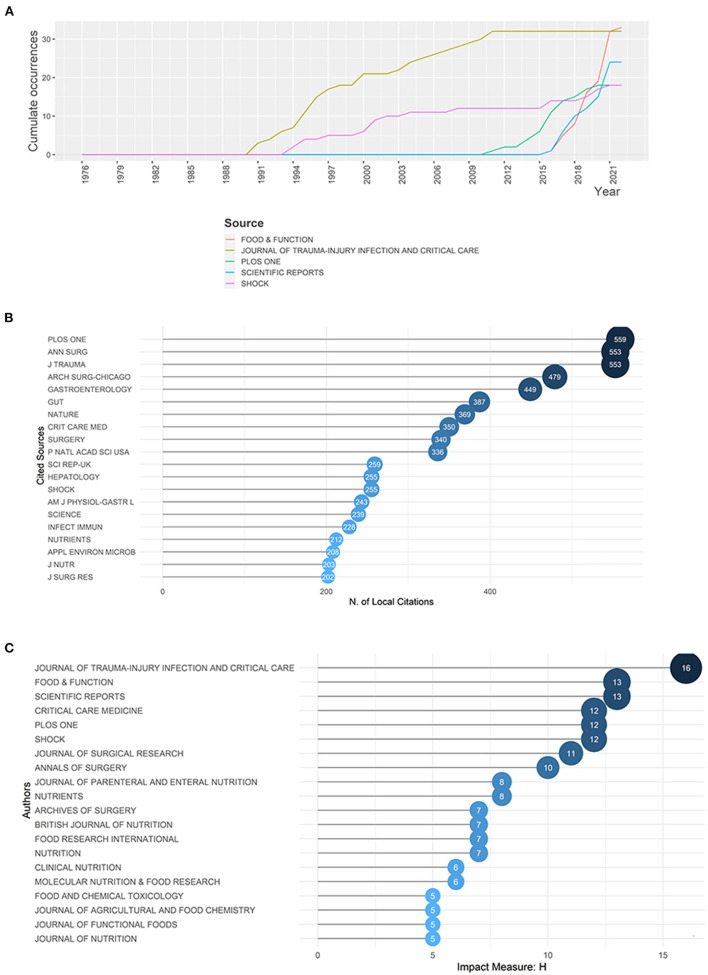
**(A)** The publications' growth of top five productive journals in gut microbiota and trauma research. **(B)** Top 20 most local cited journals in gut microbiota and trauma research. **(C)** Most local impactive journals measured by h-index in gut microbiota and trauma research.

Publications and citations were used to value the impact of 271 journals collected. The top 20 productive journals (Table 2 and Supplementary Figure S1A) published 272 documents in total from 1976 to 2022, accounting for 43.52% of all the documents. Based on Bradford's law, the top 11 productive journals were fetched to be the core journals (Supplementary Figure S1B). As presented in Table 2, Food & Function (*n* = 33) was the most prolific journal, followed by Journal of Trauma-Injury Infection and Critical Care (*n* = 32), Scientific Reports (*n* = 24), PLoS ONE (*n* = 18) and Shock (*n* = 18). Among these five journals, J Trauma was the earliest one to publish documents in this field, but its number of cumulative documents flattened beginning in 2012. By contrast, Food & Function was the latest one, with rapid growth in publication (Figure 5A).

**Table 2:** Top 20 most productive journals for burn and trauma associated with RBPs research.

**Rank**	**Sources**	**Articles**	**TCs**	**AACs**	**LCs**	**H-index**
1	Food & Function	33	413	12.52	162	13
2	Journal of Trauma-Injury Infection and Critical Care	32	1381	43.16	553	16
3	Scientific Reports	24	402	16.75	259	13
4	PLoS ONE	18	594	33.00	559	12
5	Shock	18	946	52.56	255	12
6	Journal of Functional Foods	17	89	5.24	84	5
7	Journal of Surgical Research	16	530	33.13	202	11
8	Nutrients	15	204	13.60	212	8
9	Critical Care Medicine	12	619	51.58	350	12
10	Annals of Surgery	10	1,478	147.80	553	10
11	Journal of Parenteral and Enteral Nutrition	10	478	47.80	185	8
12	Journal of Agricultural and Food Chemistry	9	103	11.44	173	5
13	Nutrition	9	210	23.33	103	7
14	British Journal of Nutrition	8	414	51.75	130	7
15	Food Research International	8	128	16.00	33	7
16	Archives of Surgery	7	463	66.14	479	7
17	Clinical Nutrition	7	147	21.00	78	6
18	Molecular Nutrition & Food Research	7	251	35.86	127	6
19	Frontiers in Microbiology	6	109	18.17	146	4
20	Journal of Nutritional Biochemistry	6	229	38.17	90	5

TCs, Total Citations; AACs, Average Article Citations.

The quantity of local citations was calculated according to reference list to measure their local impact, while total citations involved attention from other fields. In the ranking, PLoS ONE was the top of the list with 559 citations, followed by Ann Surg (553 citations) and J Trauma (553 citations) (Figure 5B). Despite having the highest output, the frequency of the local citation of Food & Function was 162, ranking 27 among all journals. In contrast, Annual of Surgery held the highest total citations (Table 2) and second-highest local citations (Figure 5B) with 10 articles in this field, indicating a high proportion of superior quality publications. When h-index was calculated to be another indication of local impact, J Trauma, Food & Function and Science Report were the top three journals (Figure 5C).

#### 3.3.2. Most impactive authors

**Figure 6:**
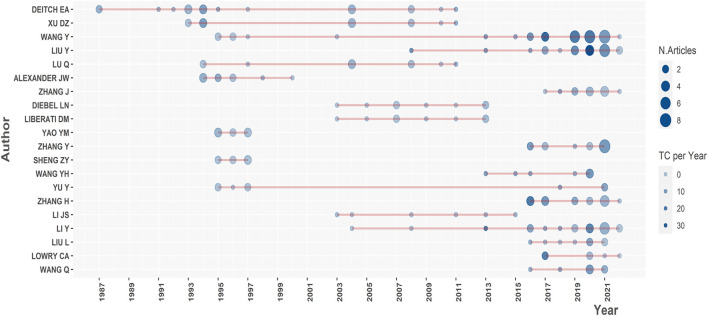
Production of the top 20 productive authors over time in gut microbiota and trauma research.

Totally, 3,991 authors published relevant articles on trauma and gut microbiota since 1976. Approximately 80% of authors posted only one article in this field by Lotka's law (Supplementary Figure S2A). On the basis of the number of documents, the top 20 relevant authors (Table 3) were identified, among whom Deitch EA was the most productive (*n* = 19), followed by Xu DZ (*n* = 12). As we further explored the author information of the articles, we discovered that all of Xu DZ' papers were written in collaboration with Deitch EA. Integrating timeline with publication, Figure 6 shows that among the top 20 relevant authors, Deitch EA was the first one to publish literature in this field and Wang Y was the most active one in the last 5 years. Not only that, but Deitch EA was the most local cited author (Supplementary Figure S2B) and the most impactive scholar measured by H-index (Supplementary Figure S2C). When it came to cooperation, Deitch EA was also the one who collaborated most frequently with others (Supplementary Figure S2D). Moreover, affiliation information of articles showed that Deitch EA was affiliated with two institutions, the Louisiana State University Medical Center and the New Jersey Medical School, which were all located in the USA, indicating the prominent position of the USA in this research field to some extent.

**Table 3:** Top 20 most productive authors for burn and trauma associated with RBPs research.

**Rank**	**Authors**	**Articles**	**TCs**	**AACs**	**LCs**	**H-index**
1	Deitch EA	19	1,271	66.89	101	15
2	Xu DZ	12	578	48.17	13	10
3	Wang Y	11	129	11.73	20	6
4	Liu Y	10	261	26.10	12	6
5	Lu Q	10	300	30.00	9	9
6	Alexander JW	9	526	58.44	10	9
7	Zhang J	9	83	9.22	8	5
8	Diebel LN	8	82	10.25	2	5
9	Liberati DM	8	82	10.25	2	5
10	Yao YM	8	192	24.00	18	7
11	Zhang Y	8	77	9.63	1	4
12	Sheng ZY	7	101	14.43	12	6
13	Wang YH	7	266	38.00	20	7
14	Yu Y	7	122	17.43	17	6
15	Zhang H	7	107	15.29	1	4
16	Li JS	6	156	26.00	1	6
17	Li Y	6	27	4.50	1	5
18	Liu L	6	84	14.00	1	4
19	Lowry CA	6	127	21.17	12	3
20	Wang Q	6	117	19.50	2	5

TCs, Total Citations; AACs, Average Article Citations; LCs, Local Citations.

### 3.4. Hotspots investigation

#### 3.4.1. Factorial analysis

The factorial analysis revealed that the top 50 high-frequency keywords showed in Supplementary Figure S3 formed two clusters (Supplementary Figures S4A, B). The blue cluster focused on the intestinal barrier in trauma patients, and the red cluster was about the gut microorganisms in trauma patients. As no documents with high citations or high contributions occurred in the blue cluster, topics concerning gut microorganisms in trauma may attract more attention than the topics regarding to intestinal barrier (Supplementary Figures S4C, D). To validate the conjecture, we added “intestinal barrier” to the research strategy, which changed research strategy to [(TS = trauma) OR (TS = traumatic)] AND [(TS = gut) OR (TS = intestin*) OR (TS = gastrointestin*) OR (TS= gastro-intestin*) OR (TS= intestinal barrier)] AND [(TS = microbiota*) OR (TS = microbiome*) OR (TS = flora) OR (TS = microflora) OR (TS = bacteria) OR (TS = prebiotic) OR (TS = probiotic)]. The visualized results of the new retrieval data were exposed in Supplementary Figures S7, S8. The growth trend of annual publication, the top 5 most prolific countries, top 5 most productive institutions, and top 5 most productive and cited journals were highly consistent with the original results (Supplementary Figures S7A–E). Deitch EA was still the most influential author (Supplementary Figures S7F, G). The visualized analysis of keywords, documents and trend topics, including the top 10 frequent keywords, co-occurrence network, historical direct citation network and trend topics' map, highly resembled the original results as well (Supplementary Figure S8). We discovered that adding “intestinal barrier” to the search terms had little effect on the search results, indicating that “intestinal barrier” was not a necessary keyword, which supported our conjecture.

#### 3.4.2. Keywords analysis

**Figure 7:**
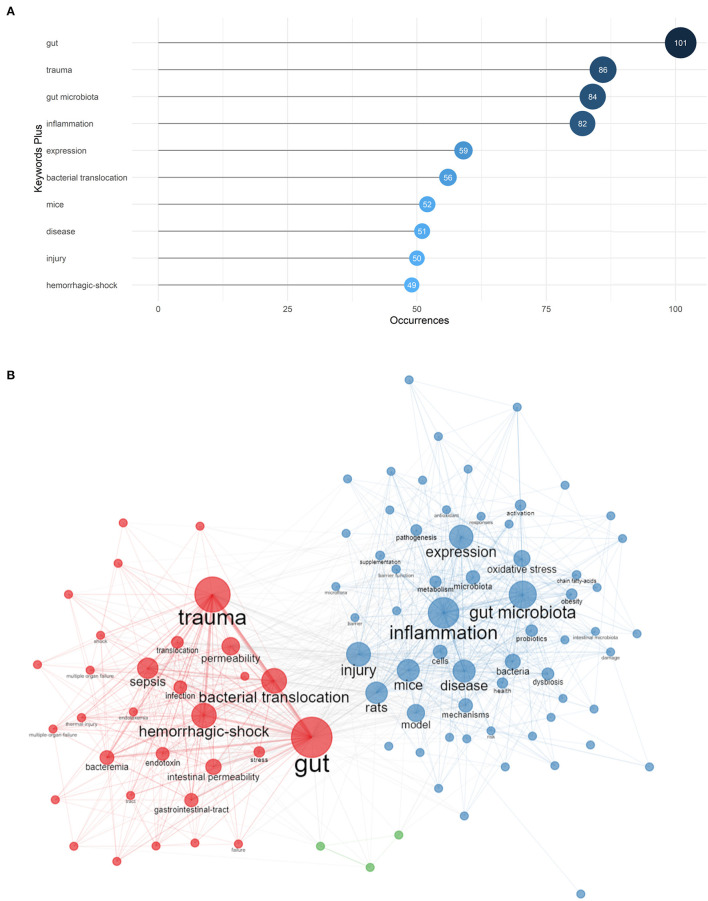
**(A)** Top 10 most frequent keywords in gut microbiota and trauma research. **(B)** The co-occurrence network of keywords which occurred at least 10 times in gut microbiota and trauma research.

As comprehensive overviews of the cores of documents, keywords can be analyzed to explore the popular themes. We retrieved 1,784 keywords from keywords plus of WoS and the top 10 frequent words were displayed in Figure 7A, comprising gut, trauma, gut microbiota, inflammation, expression, bacterial translocation, mice, disease, injury and hemorrhagic-shock.

So as to visualize the correlation between keywords, co-occurrence of network was created by Biblioshiny (Figure 7B). A co-occurrence relationship showed by link lines exists when two keywords appear in one or more documents at the same time. In addition, the more often a keyword occurs in conjunction with others, the larger its node size is. Aggregately 96 words that occurred at least 10 times were divided into three clusters (Supplementary Table S1). Cluster 1 (red), where “gut,” “trauma,” “bacterial translocation,” and “sepsis” were the largest nodes, and Cluster 3 (green), which was represented by “abdominal-trauma,” “*Escherichia coli*,” and “sepsis mortality,” were probably about the bacterial translocation in trauma as well as in complications that follow trauma. Some scholars might find gut bacterial translocation in trauma patients and further explore the influence of bacterial translocation in trauma. Cluster 2 (blue) was the largest cluster with emphasizing “gut microbiota”, “inflammation,” “expression” and “mice”. It chiefly explained the gut microbiota's impact on pathogenesis of injury, in particular the effect on inflammation, and animal models might be the main research tools.

#### 3.4.3. Documents analysis

**Figure 8:**
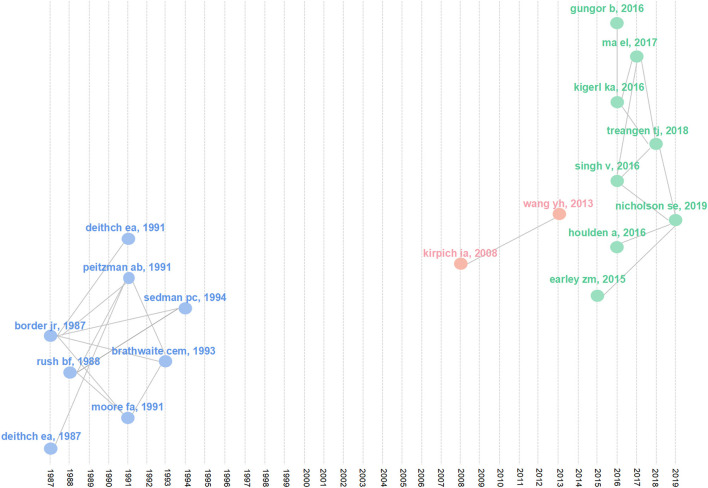
Historical direct citation network of key documents in gut microbiota and trauma research.

To profoundly investigate the main content and progress of gut microbiota and trauma studies, the top 20 local cited documents that were influential in this field were filtered out (Table 4). Connections between most of these highly cited documents were unveiled *via* the historical direct citation network (Figure 8). The link lines between articles mean that the earlier articles are cited by the latter ones. Based on these lines, the articles were distributed into 3 clusters (Table 5), pointing out three different study subjects in their respective periods. The first cluster (blue) can be derived from 1987 when scholars represented by Deitch EA (Deitch and McIntyre Bridges, 1987; Deitch et al., 1987) paid attention to bacterial translocation in trauma. The articles in the second cluster (red) discussed the therapeutical effect of probiotics in alcohol-induced liver injury (Kirpich et al., 2008; Wang et al., 2013). And the third cluster (green) covered articles on the changes and effects of gut microbiome in nervous system injury (Earley et al., 2015; Gungor et al., 2016; Houlden et al., 2016; Kigerl et al., 2016; Singh et al., 2016; Treangen et al., 2018; Wen et al., 2018; Nicholson et al., 2019). These three research subjects may represent the evolution of hotspots in gut microbiota and trauma studies.

**Table 4:** Top 20 most local cited publications for burn and trauma associated with RBPs research.

**Rank**	**Title**	**Author**	**Journal**	**Year**	**LCs**	**GCs**	**LCs/GCs ratio (%)**
1	Endotoxemia and Bacteremia During Hemorrhagic Shock The Link Between Trauma and Sepsis?	Rush BF	Ann Surg	1988	53	376.00	14.10
2	Effect of starvation, malnutrition, and trauma on the gastrointestinal tract flora and bacterial translocation	Deitch EA	Arch Surg-Chicago	1987	47	85.00	55.29
3	The Gut Origin Septic States in Blunt Multiple Trauma (ISS =40) in the ICU	Border JR	Ann Surg	1987	38	311.00	12.22
4	Gut Bacterial Translocation *via* the Portal Vein	Moore FA	J Trauma	1991	31	410.00	7.56
5	Bacterial Translocation in Trauma Patients	Peitzman AB	J Trauma	1991	23	128.00	17.97
6	Effect of stress and trauma on bacterial translocation from the Gut	Deitch EA	J Surg Res	1987	17	116.00	14.66
7	The prevalence of gut translocation in humans	Sedman PC	Gastroenterology	1994	14	264.00	5.30
8	Brain injury induces specific changes in the caecal microbiota of mice *via* altered autonomic activity and mucoprotein production	Houlden A	Brain Behavimmun	2016	14	145.00	9.66
9	Burn Injury Alters the Intestinal Microbiome and Increases Gut Permeability and Bacterial Translocation	Earley ZM	Plos One	2015	13	139.00	9.35
10	Gut dysbiosis impairs recovery after spinal cord injury	Kigerl KA	J Exp Med	2016	13	135.00	9.63
11	Moderate Traumatic Brain Injury Alters the Gastrointestinal Micro bio me in a Time-Dependent Manner	Nicholson SE	Shock	2019	13	40.00	32.50
12	The gastrointestinal tract. The “undrained abscess” of multiple organ failure	Marshall JC	Ann Surg	1993	11	346.00	3.18
13	Micro biota Dysbiosis Controls the Neuro inflammatory Response after Stroke	Singh V	J Neurosci	2016	11	242.00	4.55
14	Traumatic Brain Injury in Mice Induces Acute Bacterial Dysbiosis Within the Fecal Microbiome	Treangen TJ	Front Immunol	2018	11	43.00	25.58
15	Probiotics restore bowel flora and improve liver enzymes in human alcohol-induced liver injury: a pilot study	Kirpich IA	Alcohol	2008	10	273.00	3.66
16	Lactobacillus rhamnosus GG reduces hepatic TNFa production and inflammation in chronic alcohol-induced liver injury	Wang YH	J Nutr Biochem	2013	10	100.00	10.00
17	Endotoxin-induced bacterial translocation and mucosal permeability	Deitch EA	Crit Care Med	1991	8	127.00	6.30
18	Postinjury shock and early bacteremia. A lethal combination	Moore FA	Arch Surg-Chicago	1992	8	52.00	15.38
19	Bacterial translocation occurs in humans after traumatic injury: evidence using immunofluorescence	Brathwaite CEM	J Trauma	1993	8	83.00	9.64
20	Intestinal Microbiota in Patients with Spinal Cord Injury	Gungor B	Plos One	2016	8	65.00	12.31

GCs, Global Citations; LCs, Local Citations.

**Table 5:** Articles in historical direct citation network.

**Cluster**	**Title**	**Author**	**Journal**	**Year**	**LCs**	**GCs**
**Cluster 1**	The Gut Origin Septic States in Blunt Multiple Trauma (ISS = 40) in the ICU	Border JR	Ann Surg	1987	38	311
	Effect of stress and trauma on bacterial translocation from the Gut	Deitch EA	J Surg Res	1987	17	116
	Endotoxemia and Bacteremia During Hemorrhagic Shock The Link Between Trauma and Sepsis?	Rush BF	Ann Surg	1988	53	376
	Bacterial Translocation in Trauma Patients	Peitzman AB	J Trauma	1991	23	128
	Endotoxin-induced bacterial translocation and mucosal permeability	Deitch EA	Crit Care Med	1991	8	127
	Gut Bacterial Translocation *via* the Portal Vein	Moore FA	J Trauma	1991	31	410
	Bacterial translocation occurs in humans after traumatic injury: evidence using immunofluorescence	Brathwaite CEM	J Trauma	1993	8	83
	The prevalence of gut translocation in humans	Sedman PC	Gastroenterology	1994	14	264
**Cluster 2**	Probiotics restore bowel flora and improve liver enzymes in human alcohol-induced liver injury: a pilot study	Kirpich IA	Alcohol	2008	10	273
	Lactobacillus rhamnosus GG reduces hepatic TNFa production and inflammation in chronic alcohol-induced liver injury	Wang YH	J Nutr Biochem	2013	10	100
**Cluster 3**	Burn Injury Alters the Intestinal Micro bio me and Increases Gut Permeability and Bacterial Translocation	Earley ZM	Plos One	2015	13	139
	Gut dysbiosis impairs recovery after spinal cord injury	Kigerl KA	J Exp Med	2016	13	135
	Brain injury induces specific changes in the caecal micro biota of mice *via* altered autonomic activity and mucoprotein production	Houlden A	Brain Behavimmun	2016	14	145
	Micro biota Dysbiosis Controls the Neuroinflammatory Response after Stroke	Singh V	J Neurosci	2016	11	242
	Intestinal Microbiota in Patients with Spinal Cord Injury	Gungor B	Plos One	2016	8	65
	Bidirectional brain-gut interactions and chronic pathological changes after traumatic brain injury in mice	Ma EL	Brain Behavimmun	2017	7	56
	Traumatic Brain Injury in Mice Induces Acute Bacterial Dysbiosis Within the Fecal Microbiome	Treangen TJ	Front Immunol	2018	11	43
	Moderate Traumatic Brain Injury Alters the Gastrointestinal Microbiome in a Time-Dependent Manner	Nicholson SE	Shock	2019	13	40

Furthermore, these papers were intensively published in two periods: from 1987 to 1994 and from 2016 to 2018, which were consistent with the time when annual production began to increase, as shown in Figure 2A. Therefore, we deduced that these articles might be the origins of some meaningful themes. For instance, the article about bacterial translocation in trauma, produced by Deitch EA in 1987, was cited by other five articles in Table 4, suggesting that it may possibly be the breakthrough of the bacterial translocation study in trauma.

### 3.5. Trend topics in future

#### 3.5.1. Thematic map

In the thematic map, 3 themes were summarized from keywords plus (Supplementary Figure S5). Their centrality (abscissa) and density (ordinate) were calculated, which respectively reflected the link strength between the theme with others and the development degree of each theme. With the mean value of all the clusters' centrality and density as the origin, the coordinate axis was built. The red theme, which referred to the bacterial translocation from gut in trauma, located in the origin with the average of centrality and density, disclosing that its degree of maturity was on average. With high centrality and density, the blue theme in the first quadrant, which was related to the mechanism of the gut microbiota's impact on inflammation, was mature and had tight links with other themes, elucidating its core position in the field. The green theme, whose centrality and density were low, was in the third quadrant, which indicated the topic regarding Escherichia-coil's colonization in rats was unpopular. It's worth noting that the thematic map is dynamic, which means that the theme in the first quadrant has possibility to relocate to the second quadrant, resulting from the arising of new topics.

#### 3.5.2. Trend topics' map

**Figure 9:**
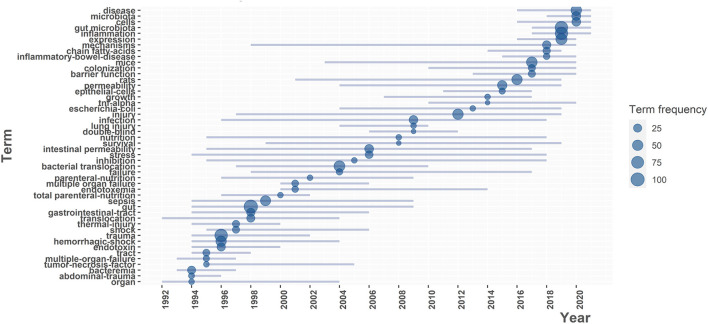
Trend Topics Analysis with the 3 keywords per year which occurred at least 10 times in the past 3 decades.

To predict trend topics in this field in the future, we exhibited some popular keywords in the past three decades (Figure 9). In the bibliometric tool, the minimum frequency of words was set to ten and the number of words per year was three. As a result, 48 keywords were contained and both their frequency and popular periods were exposed.

Keywords between 1994 and 1999 were of high density and frequency, concentrating on “gut,” “sepsis,” “trauma” and “hemorrhagic shock”. In the last century, when the mortality of trauma was higher than it is now, doctors put symptomatic treatment and decreasing lethal conditions in the first place, which may explain why scholars focus on the gut microbiota in some severe complications of trauma.

From 2000 to 2015, the frequency of new keywords decreased while the abundance increased, indicating research themes became unconcentrated in this period (Supplementary Figure S6).

In last 5 years, the typical keywords were replaced by “gut microbiota,” “inflammation” and “expression” (Figure 9), which showed that the research on the role of gut microbiota in inflammation was quite popular. The phenomenon, to some extent, can be ascribed to the rapid development of sequencing techniques such as 16S-rDNA-seq, metagenomics and single-cell RNA sequencing. These techniques can help to analyze the bacterial diversity and genetic expression quantitively, which served to explore the mechanism like inflammation response. Moreover, as the average popularity period of keywords in this map was about 12 years, this research topic might be continually prevalent in the next few years.

## 4. Discussion

Depending on a bibliometric tool to visualize data, we analyzed the literature on trauma and gut microbiota published in the past 47 years (between 1976 and 2022) to obtain a comprehensive overview of this field. As China is the most prolific country, the USA is the most impactful country. The disparity between quantity and citations of production in China implies that Chinese academics should focus more on the quality of documents in this field. Deitch EA is the scholar who possessed the most articles and the highest local citations and h-index, proving that his achievements are commonly admitted by other scholars. In journal analysis, J Trauma, Annual of Surgery and PLoS ONE are more contributive in this field. Food & Function, as a new power in this field, should not be ignored either (having had no publication for a long time, whether J Trauma will be valuable in the future is in doubt).

To seek the hotspots in this field, we combined keywords analysis, documents analysis and trend topics' map. The articles in the first cluster of the Figure 8 are all related to topic on bacterial translocation in trauma, corresponding to the core of cluster 1 (red) and cluster 3 (green) in the Figure 7B and the popular keywords in the prior period of the trend topics map (Figure 9). The highlighted words in cluster 2 (blue) of the Figure 7B coincide with the recent prevalent words in the Figure 9, indicating that the theme of cluster 2 is a hotspot at present and may remain popular in the future. Collectively, two mainstream topics were summarized: gut bacterial translocation in trauma and the impact of gut microbiota on inflammation in trauma.

### 4.1. Gut bacterial translocation in trauma

There are rare documents published between 1976 and 1986 in this field. However, in 1987, Deitch et al. demonstrated the phenomenon that trauma enhances bacterial translocation from guts to MLN (mesenteric lymph nodes) or other systematic organs in rats, especially when combined with residual necrotic tissue, which connects gut microbiota and trauma directly (Deitch et al., 1987). In Baker's animal model of hemorrhagic shock, bacterial translocation was found, and Escherichia coli was the most commonly translocated bacteria isolated from solid viscera (Baker et al., 1988), corresponding with Agalar's model (Agalar et al., 1998). In Koziol's study, bacterial translocation was also discovered in hemorrhagic shock, while Pseudomonas and Enterococcus dominated in the early stages and cultures became polymicrobial with time (Koziol et al., 1988). Subsequently, some scholars proposed that bacterial infiltration, possibly from the gut, contributes to sepsis in patients who have experienced hemorrhagic shock, based on animal experiments (Sori et al., 1988; Sorell et al., 1993). Rush et al. (1988) found that the seriously injured patients with lower blood pressure had a higher rate of positive blood cultures, and the mortality of these patients was also higher, which suggested that straight absorption of germs and endotoxin into the circulation of the traumatic and hypotensive patient plays an important role in the beginning of the syndrome that follows hemorrhagic shock. What's more, there was evidence that the occurrence of bacterial/endotoxin translocation in hemorrhagic shock might be accompanied by TNF generation, and endogenous endotoxemia may be involved in the development of multiple organ failure following shock and trauma (Yao et al., 1995). Accordingly, gut bacterial translocation appears to play an important role in some severe complications of trauma. Afterwards, more scholars flung themselves into the research of the gut bacterial translocation in trauma (Moore et al., 1991, 1992; Peitzman et al., 1991; Brathwaite et al., 1993; Sedman et al., 1994), resulting in the growth of output since 1991. Additionally, it has been reported that bacterial translocation increases the risk of postoperative infection in patients with abdominal trauma (Nieves et al., 2011; Schietroma et al., 2018). MacFie et al. (2006) reported that postoperative sepsis was more common in patients with bacterial translocation than those without (42.3 vs. 19.9%, p < 0.001), and Escherichia coli was the most common enteric organism isolated. In other perspective, a healthy gut microenvironment is also an instinctively biological barrier to resist the colonization of pathogenic bacteria. Disruption of the biological barrier probably generates infection. Earley et al. (2015) verified that burn injury altered gut microbiota and promoted the growth of γ-Proteobacteria, especially those belonging to the family Enterobacteriaceae, which contained many sepsis-related pathogens. Meanwhile, with greater α-diversity of the gut microbiome, the survival of mice with sepsis can be improved, which is possibly due to an enhanced T cell response (Cabrera-Perez et al., 2016; Fay et al., 2019). Therefore, gut bacteria may be a potential therapeutic target to improve the outcomes of patients with trauma by reducing infection. As for the mechanism of bacterial translocation in trauma, it involved the increased intestinal permeability in trauma patients, which might be related to the inflammatory response, such as the raised IL-6 level in serum (Spindler-Vesel et al., 2006; Hanscom et al., 2021). There was also a conjecture that the use of glucocorticoid might promote bacterial translocation *via* damnification of mucosal IgA synthesis (Alverdy and Aoys, 1991). However, the concretely regulatory mechanism of bacterial translocation in trauma is largely unclear and need more exploration.

### 4.2. The impact of gut microbiota on inflammation in trauma

From 2016, documents on gut microbiota and nervous system injury began to increase, which may be attributed to the establishment of the consensus that gut microbiota interacts with the nervous system and modulates the function of CNS (Cryan and Dinan, 2012; Forsythe and Kunze, 2013; Borre et al., 2014). Of the top 20 local cited documents, six refer to the gut microbiota in patients with nervous system injuries, and they were all published in 2016 and beyond. Coincidentally, the annual publications in this field have also rocketed up since 2016, with an increase in the frequency of “inflammation” and “expression.” Thus, we deduced that scholars focus more on the gut microbiota's impact on inflammation following nervous system injury than on other forms of trauma.

In 2016, Gungor et al. (2016) reported that spine core injured (SCI) patients may have a lower level of butyrate production compared with healthy subjects. Butyrate, a kind of short chain fatty acids (SCFAs), is an important metabolite created by some gut bacteria, possessing powerful anti-inflammatory action (Chen et al., 2007; Furusawa et al., 2013; Dalile et al., 2019). Moreover, gut bacterial dysbiosis caused by SCI has been found to trigger inflammation in GALT (Kigerl et al., 2016) and influence systemic and intraspinal inflammation by inducing the activation of mucosal immune cells in GALT (Dinan et al., 2013). Hence gut bacterial dysbiosis caused by SCI may in turn be related to the inflammation in SCI and affect recovery of patients. Besides SCI, bacterial dysbiosis is also found in traumatic brain injury (TBI) (Houlden et al., 2016; Treangen et al., 2018; Nicholson et al., 2019; Urban et al., 2020; Opeyemi et al., 2021). Houlden et al. observed the increased noradrenaline (NE) release in the caecum of brain-injured mice models, as well as the positive correlation between the abundance of Peptococcaceae and RANTES (CCR5) level, a type of proinflammatory cytokine, in the gut tissue (Houlden et al., 2016). The responses of proinflammatory Th1 and Th17 elicited by bacterial dysbiosis and the link between bacterial dysbiosis and degenerative outcome in mice with massive brain infarction were also observed (Singh et al., 2016). It has also been reported that LPS, a kind of toxic bacterial component, can induce the activation of microglia, which then aggravates neuroinflammation in TBI (Wen et al., 2018). What's more, You et al. (2022) discovered the decreased level of bile acid, especially the secondary bile acid, in feces and plasma after TBI, which might be related to the gut bacteria such as Staphylococcus and Lachnospiraceae. Then the deficiency in secondary bile acids might boost intestinal inflammation in patients (Sinha et al., 2020). These findings demonstrated the presence of bacterial dysbiosis in neurotrauma as well as the critical role of gut microbiota in inflammation. As the study of this theme hasn't been carried out for a long time, the specific pathways by which gut microbiota affect the pathogenesis of neurotrauma are still not entirely clear and need more exploration.

Not only the mechanisms of the impact of gut bacteria on trauma but also the methods that can alter gut microbiota have attracted people's attention. Two of the most local cited documents documented the functions of probiotic supplements in treatment of alcohol-induced liver injury, including suppressing inflammation (Kirpich et al., 2008; Wang et al., 2013). Meanwhile, the supplementation of probiotic Lactobacillus rhamnosus GG (LGG) might improve intestinal integrity by inhibiting miR122a, which resulted in occludin restoration, and increasing mRNA expression of tight junction (TJ) proteins in mice with alcohol-induced liver injury (Zhao et al., 2015; Chen et al., 2016). The positive effect of probiotic treatment has been documented in SCI as well, including locomotor recovery and regulating T lymphocyte activation (Kigerl et al., 2016). In serious TBI, with probiotic treatment, the deviated Th1/Th2 response might be mitigated, which then could lower the risk of nosocomial infections (Tan et al., 2011). Moreover, fecal microbiota transplantation (FMT), another method to alter gut microbiota, was found to increase the production of SCFAs and suppress the IL-1/NF-κB signaling in the spinal cord and NF-κB signaling in the intestine after SCI (Jing et al., 2021). FMT was also reported to relieve neurological deficits after TBI through TMA-TMAO-MsrA signaling pathway (Du et al., 2021). Some specific bacteria had also been shown to have neuroprotective effects through different mechanisms in mice with TBI (Li et al., 2018; Ma et al., 2019). What's more, there are experiments verifying that dietary structures such as high protein and supplements of specific foods like rice bran phenolic extract, oolong tea and so on can lead to changes in the gut microbiota and aggravation or relief of inflammation in injuries (Zhang et al., 2014; Power et al., 2016; Feng et al., 2020; Xiao et al., 2020; Ge et al., 2021; Li et al., 2021; Snelson et al., 2021). Most of these experiments were conducted in animal models, which means that we need more clinical evidence to support the therapeutic functions of these methods.

With the development of high-throughput sequencing technology, researchers are able to directly explore the changes and functions of the microorganisms in trauma, which may partly contribute to the result that topics concerning gut microorganisms in trauma may attract more attention than the topics regarding to intestinal barrier, in factorial analysis. Moreover, here we extrapolate that with the continual update of research techniques, such as single-cell RNA sequencing, spatial transcriptome sequencing and multi-omics analysis, scholars can further disclose the concrete regulatory networks and pathways induced by microbiota in the mechanism of trauma development, which may be a direction for future research in this field. The studies on the therapies will also continue and more clinical trials will be carried out.

To our knowledge, this study is the first biliometric analysis involving the influence of gut microbiota in trauma patients. Nevertheless, there are still some limitations. Firstly, bibliometrics can't appraise the quality, rationality, and limitations of the articles that we analyzed. Thus, the study can't replace some other review types, such as systematic review and meta-analysis. Secondly, we retrieved articles that were recorded in the Web of Science Core Collection (WoSCC) by May 15, 2022. Due to the restriction of retrieval and analysis technology, WoSCC was the only database we utilized to download publication information. Therefore, the related articles, which were not in the database, and some recently published researches, were not included in the study. Thirdly, due to the limitation of the retrieval method, we didn't distinguish basic and clinical researches and we paid more attention to conclusions of the articles. Last but not least, as we tended to analyze the articles with high citations, some cutting-edge articles with low citations might be ignored, resulting in the hysteresis quality of the study to some extent.

## 5. Conclusion

We overviewed the research on gut microbiota and trauma from a bibliometric standpoint in this study. This field is experiencing a stage of rapid growth and gaining incremental attention. China and the USA are the major producers and leaders in this field. The University of Colorado and Deitch EA are the most prolific institution and author, respectively. As Food & Function published the most documents, PLoS ONE and J Trauma are the most local journals, separately measured by local citations and h-index. Paying more attention to them can help us keep abreast of the current trends in this field. Our study identified two mainstream topics: bacterial translocation in trauma and the impact of gut microbiota on inflammation in trauma, and both of them are related to the prognosis of trauma patients. Due to the faultiness of the explanation of how gut microbiota translocate to parenteral tissues in trauma and how they mediate the development of inflammation, more exploration is required so that new treatment targets can be found. Besides, the topic concerning therapeutic functions of methods which can alter gut microbiota, starts to develop. We hope that the fruits of further research will be translated into medical therapies in the near future, and this study can be a helpful reference for scholars in this field.

## Data availability statement

The original contributions presented in the study are included in the article/Supplementary material, further inquiries can be directed to the corresponding author.

## Author contributions

Conception/design, collection and/or assembly of data, data analysis and interpretation, manuscript writing, and final approval of manuscript: RH, YLi, MJ, YLu, MZ, SX, ZC, WZ, JL, XT, SW, YZ, JH, LJ, MG, ZH, MW, and SJ. All authors contributed to the article and approved the submitted version.
