# Supplementary material for: A bibliometric analysis of the role of microbiota in trauma
Source: Front Microbiol. 2023 Feb 2;14:1091060. doi: 10.3389/fmicb.2023.1091060 (PMC9932281; doi:10.3389/fmicb.2023.1091060)
Supplement: Supplementary file 1 [file Data_Sheet_1.PDF]

Supplementary Figures

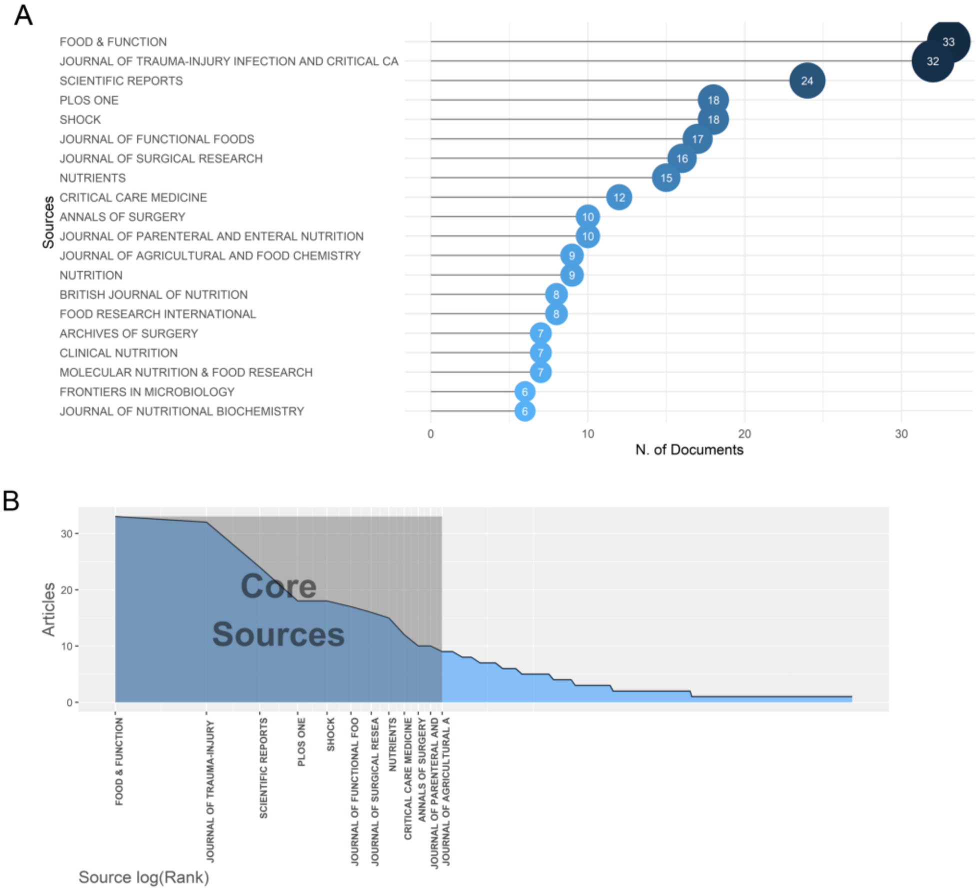

Figure S1. Supplementary materials for journal analysis. (A) Relevant journals in gut microbiota and trauma research. **(B)** Core journals based on Bradford's law in gut microbiota and trauma research.

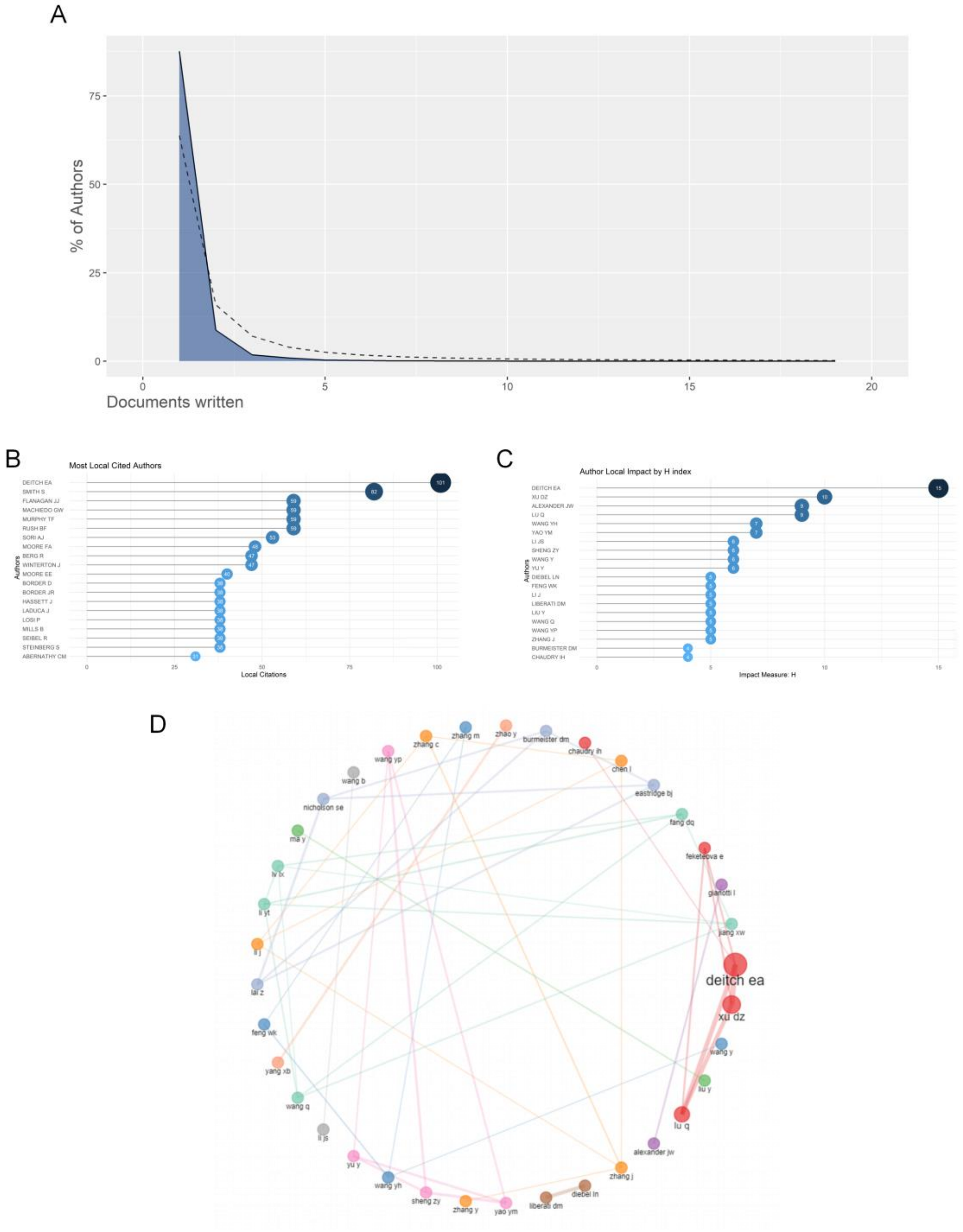

Figure S2. Supplementary materials for author analysis. (A) The frequency distribution of scientific productivity in gut microbiota and trauma research by Lotka's law. (B) Top 20 most local cited authors in gut microbiota and trauma research. (C) Top 20 local impactive authors measured by H-index in gut microbiota and trauma research. (D) Collaboration network of the top 20 productive authors in gut microbiota and trauma research.

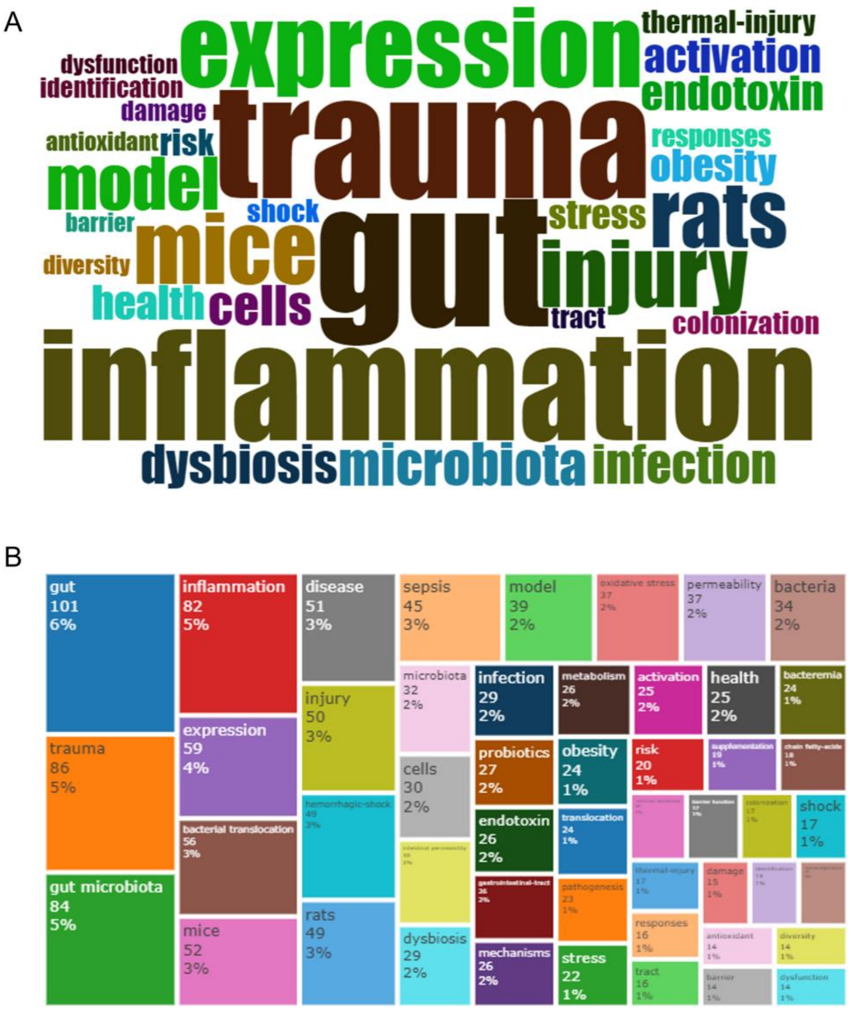

Figure S3. Supplementary materials for keyword analysis. (A) WordCloud of the top 50 frequent keywords in gut microbiota and trauma research. **(B)** TreeMap of the top 50 frequent keywords in gut microbiota and trauma research.

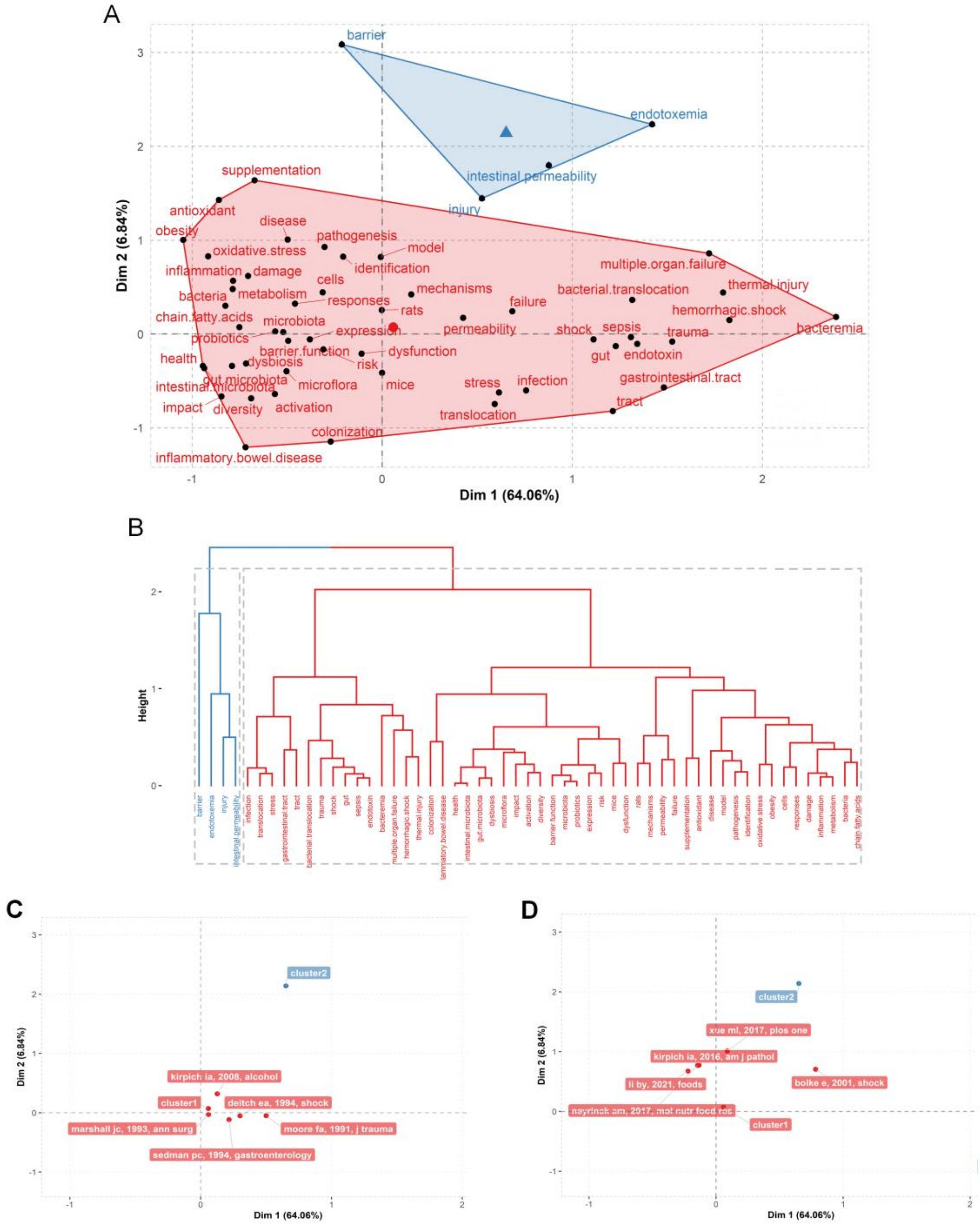

Figure S4. Factorial analysis. (A) Conceptual structure map of keywords with method of MCA in gut microbiota and trauma research. **(B)** Topic dendrogram in gut microbiota and trauma research. **(C)** Factorial map of documents with the most cited in gut microbiota and trauma research. **(D)** Factorial map of documents with the highest contributions in gut microbiota and trauma research.

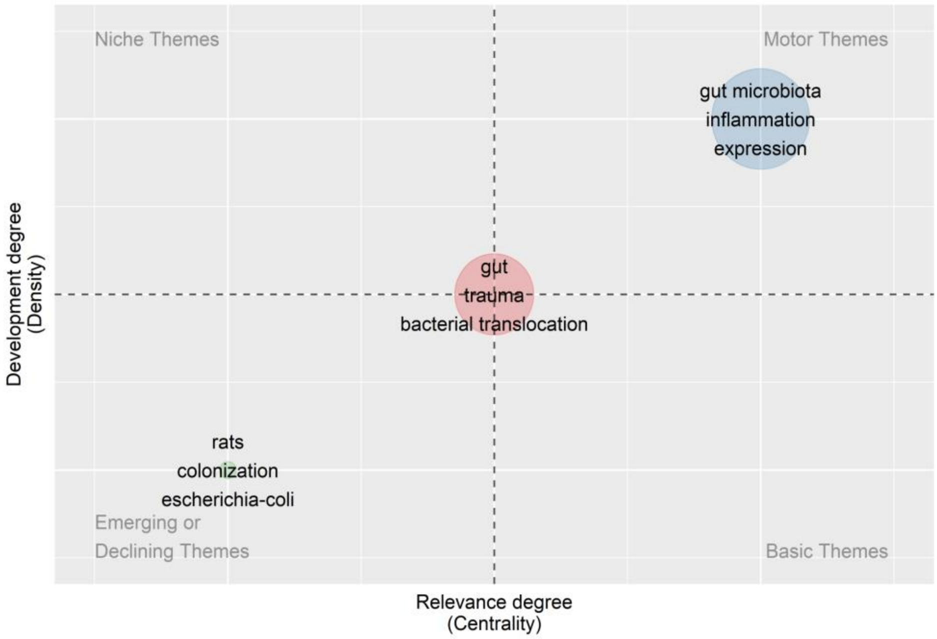

Figure S5. Thematic map of gut microbiota and trauma research, distributing themes into four quadrants which are respectively defined as motor themes, niche themes, emerging or declining themes and basic themes. The abscissa represents relevance degree (centrality) and the ordinate indicates development degree (density).

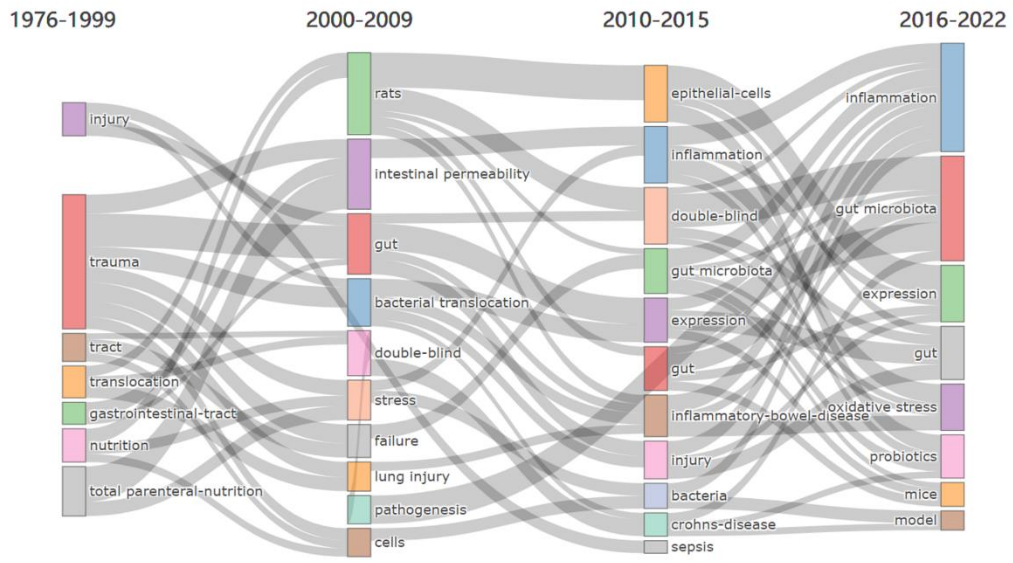

Figure S6. Thematic evolution with three cutting points of year 1999, 2009, and 2015 in gut microbiota and trauma research.

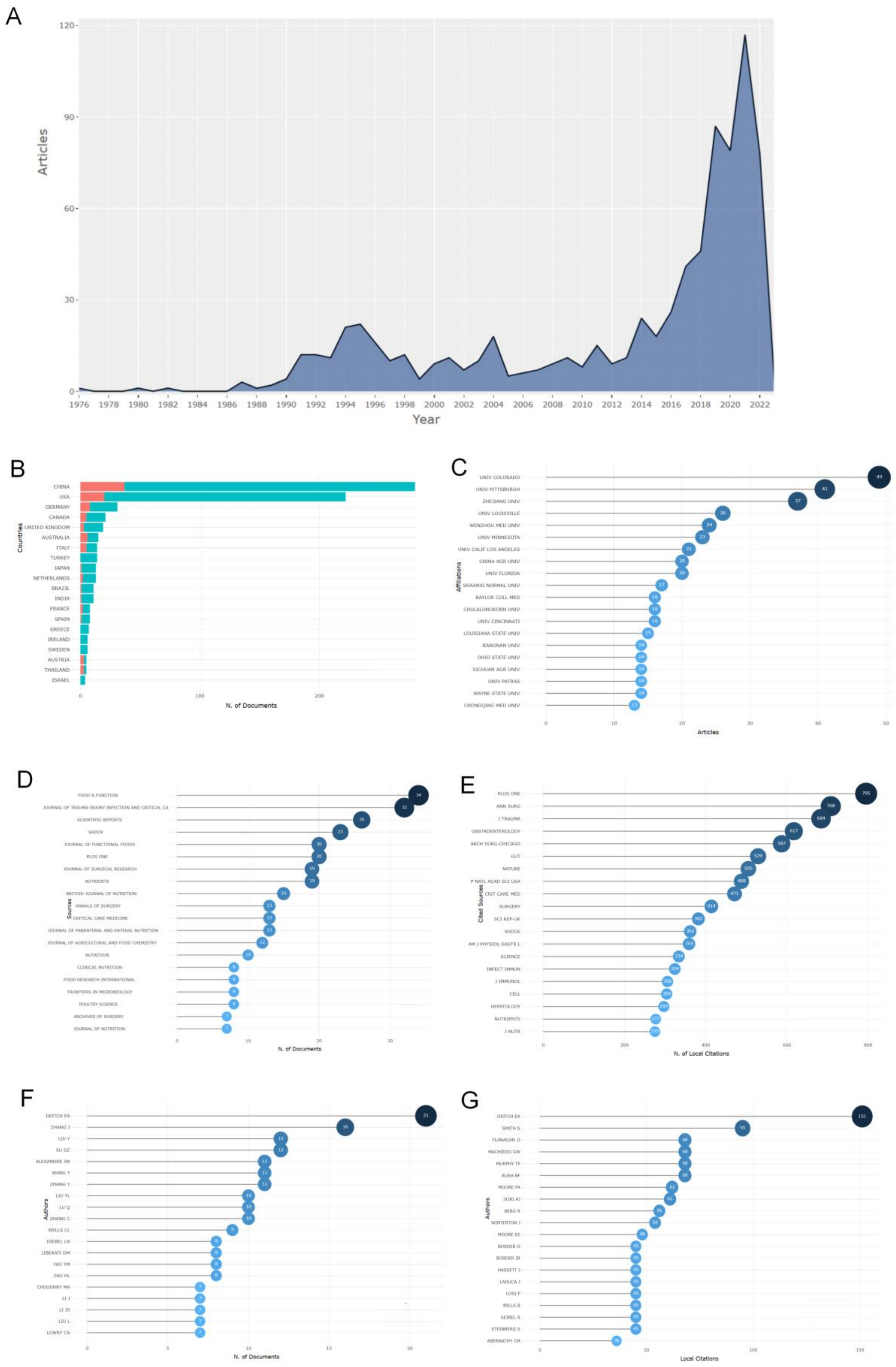

Figure S7. Visualized results of the research strategy with the addition of "intestinal barrier"

(A) Quantity of annual scientific production in gut microbiota and trauma research from 1976 to 2022. **(B)** Top 20 countries production and collaboration histogram based on the nationality statistics of the corresponding authors in gut microbiota and trauma studies. Abbreviations: SCP, single country publications. MCP, multiple country publications. **(C)** Top 20 most productive institutions in gut microbiota and trauma research. **(D)** Relevant journals in gut microbiota and trauma research. **(E)** Top 20 most local cited journals in gut microbiota and trauma research. (G)Top 20 most local cited authors in gut microbiota and trauma research.

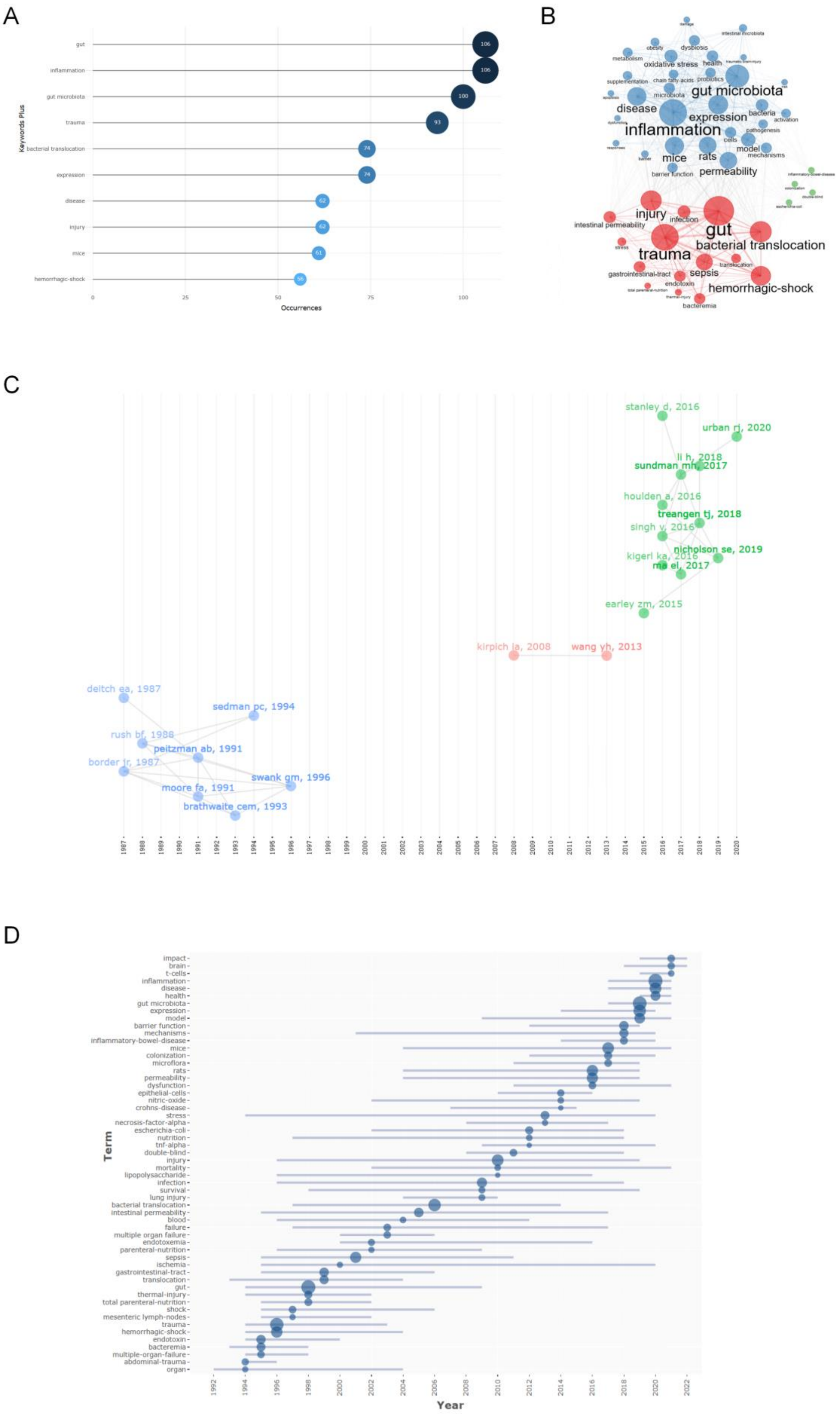

Figure S8. Visualized results of the research strategy with the addition of "intestinal barrier"
(A) Top 20 most frequent keywords. **(B)** The co-occurrence network of keywords which occur at

least 10 times in gut microbiota and trauma research. **(C)** Historical direct citation network of key documents in gut microbiota and trauma research. **(D)** Trend Topics Analysis with the 3 keywords per year which occurred at least 10 times in the past 3 decades.

Table S1. The information of the keywords which occurred at least 10 times in gut microbiota and trauma research.

Node	Cluster	Betweenness	Closeness	PageRank
gut	1	685.3760905	0.007462687	0.057284725
trauma	1	289.2466927	0.006944444	0.045539089
bacterial translocation	1	74.27090695	0.006369427	0.027422115
hemorrhagic-s hock	1	69.934418	0.006289308	0.028749066
sepsis	1	69.1121406	0.006493506	0.022370225
permeability	1	58.43126905	0.006410256	0.01829042
intestinal permeability	1	40.68403832	0.00617284	0.015619683
infection	1	17.96470073	0.005847953	0.011328085
endotoxin	1	19.60533153	0.005747126	0.013132544
gastrointestinal-tract	1	8.847337834	0.005780347	0.013322668
bacteremia	1	8.781162119	0.005649718	0.016081737
translocation	1	51.10860446	0.005988024	0.012314668
stress	1	47.35510851	0.005952381	0.010671312
shock	1	5.743404873	0.005181347	0.007776267
thermal-injury	1	0.378905227	0.005154639	0.008123909
tract	1	1.938137576	0.005376344	0.007815637
multiple organ failure	1	1.218346655	0.005376344	0.00869752
endotoxemia	1	4.138656635	0.005376344	0.008214862
failure	1	1.495473495	0.005263158	0.005582984
multiple-organ-failure	1	3.525701686	0.005263158	0.007955477
inhibition	1	3.8417507	0.005494505	0.005351494
lung injury	1	0.225097422	0.005376344	0.005350687
multiple-organ failure	1	0.089401668	0.005050505	0.005439246
nutrition	1	0.675916633	0.005291005	0.003749087
organ	1	2.566249498	0.005319149	0.006879845
parenteral-nutrition	1	1.05160574	0.005076142	0.004672673
total parenteral-nutrition	1	0.719146904	0.005102041	0.004459434
tumor-necrosis-factor	1	0.170314024	0.004950495	0.004521658
blood	1	0.567480292	0.005128205	0.004992107
mesenteric lymph-nodes	1	0.134188603	0.004975124	0.004479452
survival	1	0.29502411	0.004739336	0.003460027
gut microbiota	2	423.8415862	0.007092199	0.032070125
inflammation	2	378.4482158	0.007246377	0.038686262
expression	2	342.404796	0.006993007	0.028313893
mice	2	191.3580035	0.006849315	0.02409081
disease	2	186.5311792	0.006756757	0.025869817
injury	2	205.540927	0.006993007	0.028776919
rats	2	158.0522144	0.006711409	0.023263795
model	2	111.6053885	0.006535948	0.017963038
oxidative stress	2	43.76575743	0.006134969	0.017176423
bacteria	2	48.32527088	0.00617284	0.016078868

microbiota	2	70.12546027	0.006097561	0.014350253
cells	2	67.96835973	0.006097561	0.013561243
dysbiosis	2	25.47153844	0.005813953	0.011538932
probiotics	2	44.85658178	0.006060606	0.011954674
mechanisms	2	20.07736591	0.006024096	0.012609722
metabolism	2	25.83123784	0.005952381	0.01106189
activation	2	29.71896992	0.00591716	0.011520716
health	2	11.90597065	0.005813953	0.010596302
obesity	2	7.128691399	0.005649718	0.010488562
pathogenesis	2	21.1298676	0.00591716	0.010481691
risk	2	111.7332812	0.005617978	0.00766126
supplementation	2	8.46394867	0.005586592	0.00811097
chain fatty-acids	2	13.05517542	0.005747126	0.00893938
intestinal microbiota	2	5.345417089	0.005405405	0.00646689
barrier function	2	13.75625521	0.005813953	0.008914065
colonization	2	97.98598315	0.005102041	0.005893053
responses	2	6.463768529	0.005555556	0.006412488
damage	2	13.02748386	0.005555556	0.00786846
identification	2	0.183279017	0.005263158	0.003939087
antioxidant	2	10.38285025	0.005376344	0.006858981
barrier	2	2.551749832	0.005405405	0.00674692
diversity	2	8.430197774	0.00462963	0.004950321
dysfunction	2	1.33247141	0.005291005	0.004743868
impact	2	2.658681202	0.004830918	0.004103919
inflammatory-bowel-disease	2	2.107951051	0.004830918	0.003879416
microflora	2	7.463790217	0.005464481	0.005588296
epithelial-cells	2	0.111664848	0.005319149	0.003975691
in-vitro	2	0.222652727	0.005050505	0.004075418
acid	2	0.079329417	0.005208333	0.003929671
butyrate	2	3.711278207	0.005464481	0.006584957
modulation	2	3.421305905	0.005208333	0.005030223
nf-kappa-b	2	6.085398023	0.005050505	0.006447178
prevention	2	0.968431119	0.005235602	0.004106988
protects	2	0.319354882	0.005464481	0.005330216
system	2	0.358284867	0.004901961	0.003622446
axis	2	0.834772797	0.005	0.004116103
brain	2	1.473319533	0.005	0.004055073
growth	2	2.393837007	0.005076142	0.004244599
gut microbiome	2	0	0.003676471	0.001996638
receptor	2	2.746402094	0.005076142	0.005069726
traumatic brain-injury	2	0.641993167	0.004950495	0.003843891
apoptosis	2	0.030307785	0.005025126	0.003090634
association	2	0.505399863	0.004347826	0.003068194
double-blind	2	0	0.003448276	0.002188637

ethanol	2	3.040498879	0.005617978	0.006876906
growth-performance	2	0.022160665	0.004830918	0.004079774
insulin-resistance	2	0.543322487	0.005181347	0.003880892
liver	2	0.635026063	0.005291005	0.005195847
mouse model	2	0.624941697	0.005464481	0.005009995
nitric-oxide	2	4.472317431	0.005208333	0.004695569
tnf-alpha	2	3.454456488	0.005405405	0.004726437
ulcerative-colitis	2	0.544289044	0.004366812	0.003144349
abdominal-trauma	3	2.620073635	0.005076142	0.006451506
escherichia-coli	3	13.13613412	0.005319149	0.004723517
septic morbidity	3	1.480777779	0.004901961	0.005258914
